# A Variant in Abhydrolase Domain Containing 14A (*ABHD14A*) and the Suspicion of Aminoacylase 1 (*ACY1*) Deficiency

**DOI:** 10.1002/jmd2.70124

**Published:** 2026-09-18

**Authors:** Lil Klaas, Estrella López‐Martín, Beatriz Martinez‐Delgado, Manuel Posada de la Paz, Antonia Ribes, Jörn Oliver Sass

**Affiliations:** ^1^ Research Group Inborn Errors of Metabolism, Department of Natural Sciences, Institute for Functional Gene Analytics (IFGA) Bonn‐Rhein‐Sieg University of Applied Sciences Rheinbach Germany; ^2^ Instituto de Investigación de Enfermedades Raras (IIER), Instituto de Salud Carlos III (ISCIII) Madrid Spain; ^3^ CIBER of Rare Diseases (CIBERER) Instituto de Salud Carlos III (ISCIII) Madrid Spain; ^4^ Institut de Investigacions Biomèdiques August Pi‐Sunyer (IDIBAPS), Hospital Clínic University of Barcelona Barcelona Spain

**Keywords:** abhydrolase domain containing 14A (ABHD14A), ACY1, aminoacylase 1 deficiency, inborn error of metabolism, non‐mediated RNA decay (NMD), read‐through transcript

## Abstract

Aminoacylase 1 (ACY1) deficiency is a rare autosomal recessive inborn error of metabolism, characterised by accumulation of *N*‐acetyl‐l‐amino acids in urine and highly variable clinical presentations. In contrast, the abhydrolase domain containing 14A (*ABHD14A*) has been suggested to play a role in various metabolic processes and may be linked to diseases, although its precise function remains unclear. Importantly, ABHD14A is known to form a read‐through transcript with ACY1, indicating the possibility that variants in ABHD14A may influence ACY1‐related phenotypes. Here, we report a patient with suspected ACY1 deficiency, carrying a heterozygous variant in the *ACY1* gene (c.1178G>A) together with a variant in *ABHD14A* (c.95G>A). The aim of this study was to functionally assess ACY1 activity and to investigate whether the *ABHD14A* variant affects ACY1 expression or enzyme activity. To this end, ACY1 enzyme activity was measured in patient‐derived lymphocytes, and overexpression experiments in HEK293 cells were performed to analyse ACY1 enzyme activity as well as mRNA and protein levels of the patient‐specific variants. We show that the patient‐derived variant in *ABHD14A* does not affect ACY1 expression or enzyme activity. In addition, our report provides the first experimental evidence that the *ABHD14A*–*ACY1* read‐through does not lead to a functional detectable protein, supporting the hypothesis of nonsense‐mediated RNA decay.

## Synopsis

Variants in *ABHD14A* and *ACY1* that have been identified in a patient revealed preserved ACY1 enzyme activity, thus providing experimental evidence to support nonsense‐mediated RNA decay of the ABHD14A–ACY1 read‐through transcript rather than an impact of altered *ABHD14A* on ACY1.

## 1 Introduction

**FIGURE 1:**
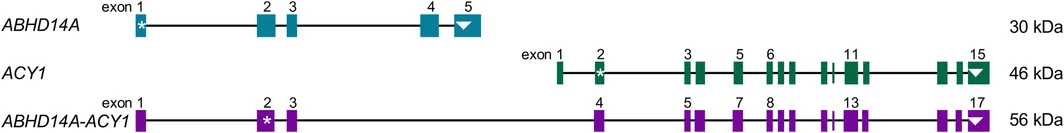
Genomic sequence of *ABHD14A*, *ACY1*, and *ABHD14A*–*ACY1* on chromosome 3p21.1. The read‐through product *ABHD14A*–*ACY1* is almost identical in the *ACY1* sequence, but the N‐terminal region differs due to an alternative start codon resulting in a shortened *ABHD14A* sequence in the read‐through. *: start of translation; 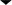: stop of translation.

*ACY1*, the gene that encodes aminoacylase 1 (ACY1; EC 3.5.1.14), is located on chromosome 3p21.1 and encodes a 408‐amino acid protein (46 kDa) in the human (Figure 1) [1]. ACY1 is widely expressed in human tissues, particularly in kidney and brain [2, 3], and catalyses the hydrolysis of various *N*‐acetyl‐l‐amino acids [4, 5]. Aminoacylases are zinc‐binding metalloenzymes classified within the M20 family and have been conserved throughout evolution [4, 6]. ACY1 has predominantly been described as a cytosolic enzyme, although alternative subcellular localisations have also been proposed [7]. Homozygous and compound‐heterozygous variants in the *ACY1* gene can cause ACY1 deficiency, a rare inborn error of metabolism, characterised by increased urinary levels of *N*‐acetylated amino acids which are detectable by organic acid analysis [2, 8]. To date, fewer than 30 individuals with genetic ACY1 deficiency have been reported, with a wide spectrum of clinical features; asymptomatic individuals are also known [9]. ACY1 is increasingly linked to various cellular processes and diseases, and altered ACY1 expression has been associated with several cancer types. For instance, increased ACY1 expression may promote the progression of non–small cell lung cancer (NSCLC) [10]. Similarly, increased ACY1 expression was detected in rectal cancer tissues and cells, where reduced ACY1 levels inhibited cellular proliferation and induced apoptosis [11]. In contrast, earlier studies demonstrated that reduced or absent ACY1 expression was observed in subsets of small cell lung cancer [12], suggesting that ACY1 may have tissue‐dependent functions. Moreover, ACY1 has been proposed as a potential biomarker in Type 2 diabetes [13].

However, little is known about detailed cellular functions of ACY1 and the potential contribution of interacting or neighbouring genes, such as *ABHD14A*. The gene encoding the human abhydrolase domain containing 14A (*ABHD14A*) is also located on chromosome 3p21.1. It comprises five exons (Figure 1) [14]. ABHD14A is a 271‐amino acid protein (30 kDa) that is predicted to possess hydrolase activity and belongs to the family of α/β hydrolase domain (ABHD) proteins, a group of enzymes predicted to be involved in lipid metabolism and signal transduction [15]. The ABHD family consists of approximately 19 proteins, and they are a part of a superfamily that possesses an α/β‐hydrolase fold [16]. *ABHD14A* has repeatedly been described as a gene of potential relevance in neurodevelopment; however, its function is poorly understood [17]. Its mouse ortholog *Dorz1* was suggested to be involved in the development of granule neurons in the cerebellum [18]. Like *ACY1*, some studies have proposed *ABHD14A* as a candidate gene in autism spectrum disorders [19, 20, 21]. Despite its evolutionary conservation, ABHD1A remains functionally poorly characterised, and its biochemical function is still unknown [17].

In addition to the individual genes, a naturally occurring read‐through transcript spanning the neighbouring genes *ABHD14A* and *ACY1* at the chromosome 3p21.1 locus has been described [22]. This transcript encodes a sequence that is largely identical to the sequence of ACY1, while the sequence of ABHD14A in this transcript differs at the N‐terminus due to the use of an alternative start codon upstream of *ACY1* (Figure 1). To date, the co‐occurrence of variants in *ACY1* and *ABHD14A* has not been described in any human. It is still unknown whether *ABHD14A* and *ACY1* are functionally connected and whether *ABHD14A* variants will modulate ACY1 activity or any other biological effects linked to ACY1.

Now, the identification of a patient with heterozygous variants in both *ABHD14A* and *ACY1* has prompted systematic studies for possible effects of *ABHD14A* alterations on ACY1, thus investigating the functional relevance of the ABHD14A–ACY1 read‐through and its potential contribution to the pathophysiology of ACY1 deficiency.

## 2 Patient, Materials and Methods

### 2.1 Patient

The patient is an 11‐year‐old girl at the time of the genetic study, born at term after a pregnancy with gestational diabetes. Early psychomotor development was delayed, with independent walking achieved at 16 months and persistent global developmental delay. Epilepsy began at 5 months of age with epileptic spasms, later evolving into drug‐resistant focal epilepsy with multiple seizure types, predominantly during sleep, including brief tonic spasms, focal motor seizures with head deviation, generalised chronic seizures, and episodes of impaired awareness. Brain MRI showed multifocal white matter abnormalities with focal areas of demyelination and gliosis. Serial EEGs demonstrated persistent left parieto‐occipital epileptiform abnormalities with focal seizures. Extensive metabolic investigations, thyroid function, lysosomal enzyme testing, nerve conduction studies, visual evoked potentials, and ophthalmological examination were unremarkable. There was no family history of intellectual disability or epilepsy. She had a healthy 3‐year‐old brother. At this moment, the patient remains undiagnosed.

### 2.2 Cell Culture, Transfection and Immortalisation

Human embryonic kidney (HEK293) cells were cultivated as previously described [23]. For the expression constructs, the cDNA of human ACY1 (NM_000666.3), human ABHD14A (NM_015407.5), human ABHD14A–ACY1 (NM_001316331.2) and their variants were cloned into the mammalian expression vector pcDNA3.1(+) and were obtained from GenScript. To obtain cell lines stably overexpressing ACY1, ABHD14A and their variants, transfections of pcDNA3.1(+) constructs in HEK293 cells were performed using Lipofectamine 3000 (Invitrogen) according to the manufacturer's instructions. As a negative control, the empty pcDNA3.1(+) vector was used. Forty‐eight hours post transfection, G418 (Carl Roth) was used for selection of successfully transfected cells. Lymphocytes were isolated from patient‐derived whole blood samples by density gradient centrifugation using BioColl separating solution according to the manufacturer's instructions and subsequently immortalised using Epstein–Barr virus. Lymphocytes were grown in Roswell Park Memorial Institute (RPMI) 1640 medium (Invitrogen) supplemented with 20% foetal bovine serum (Invitrogen), 100 units penicillin G/mL and 100 μg streptomycin sulphate/ml at 37°C and 5% CO_2_.

### 2.3 Enzyme Activity Assay and Mutation Analysis

ACY1 enzyme activity assays were performed using homogenised cell sediments from patient‐derived Epstein–Barr virus‐transformed lymphocytes or HEK293 cells stably transfected with the expression constructs. Lymphoblastoid cells derived from an individual with proven ACY1 deficiency served as positive control. Enzyme activity was assessed using a ninhydrin‐based photometric assay described previously [24]. The DNA extracted from peripheral blood of the entire family was analysed using whole‐exome sequencing (WES). WES of the trio was performed with the Nextera Truseq Rapid Enrichment kit (Illumina) and sequenced in a NextSeq500 Illumina sequencer using High Output Flow Cell 2 × 75 cartridges (Illumina). WES data were analysed using the RD‐Connect Genome‐Phenome Analysis Platform (GPAP), a web‐based environment developed for rare disease diagnosis and research [25]. Variants were annotated with population frequency, predicted functional consequence, gene–disease associations, in silico pathogenicity scores, and available clinical classifications from public databases. Variant filtering and prioritisation were performed by integrating molecular data with the patient's phenotype encoded using Human Phenotype Ontology (HPO) terms. Filtering strategies included allele frequency, predicted impact on protein function or splicing, inheritance model (including de novo, dominant, homozygous, and compound heterozygous variants), or consistency with the reported clinical phenotype. Candidate variants were assessed according to the recommendations of the American College of Medical Genetics and Genomics and the Association for Molecular Pathology (ACMG/AMP), considering available clinical, genetic, and functional evidence. The final interpretation was based on multidisciplinary expert review, and clinically relevant variants were further explored. Sanger sequencing was performed to confirm the candidate variants. In silico assessment of the identified sequence variants was done by using GeneBe [26] and tools listed in the GeneBe report.

### 2.4 Protein Quantitation and Immunoblotting

To determine protein concentrations, a Lowry assay was performed [27]. Proteins were separated by SDS‐PAGE and transferred onto PVDF membranes (Bio‐Rad). Immunoblotting was performed using the following antibodies: rabbit anti‐ACY1 (GeneTex GTX110348, 1:1000), rabbit anti‐ABHD14A (Invitrogen PA5‐46077, 1:1000), and mouse anti‐α‐tubulin (Abcam ab7291, 1:5000). Detection of protein bands was performed using SuperSignal West Femto maximum sensitivity substrate (Thermo Scientific).

### 2.5 Quantitative Real‐Time PCR (qPCR)

Total RNA was extracted from cell sediments using the NucleoSpin RNA blood kit (Macherey‐Nagel) according to the manufacturer's instructions. A total of 1 μg of RNA was reverse transcribed using SuperScript IV reverse transcriptase (Invitrogen) and an oligo(dT) primer according to the manufacturer's instructions. The Blue S'Green qPCR Kit (Biozym) was used to perform qPCR. The primers used are listed in Table S1. qPCR was performed using a LightCycler qTower [2] (Analytik Jena). The thermal cycling conditions were as follows: denaturation at 95°C for 2 min followed by 40 cycles of amplification at 95°C for 5 s and 63°C for 20 s. Fluorescence was detected during annealing at 520 nm. Melting curve analysis was performed after PCR. Relative mRNA expression levels were calculated using the ΔΔ*C*_t_ method, using the housekeeping gene *GAPDH* as a reference [28].

## 3 Results

### 3.1 Genetic Analysis and Organic Acid Analysis

**FIGURE 2:**
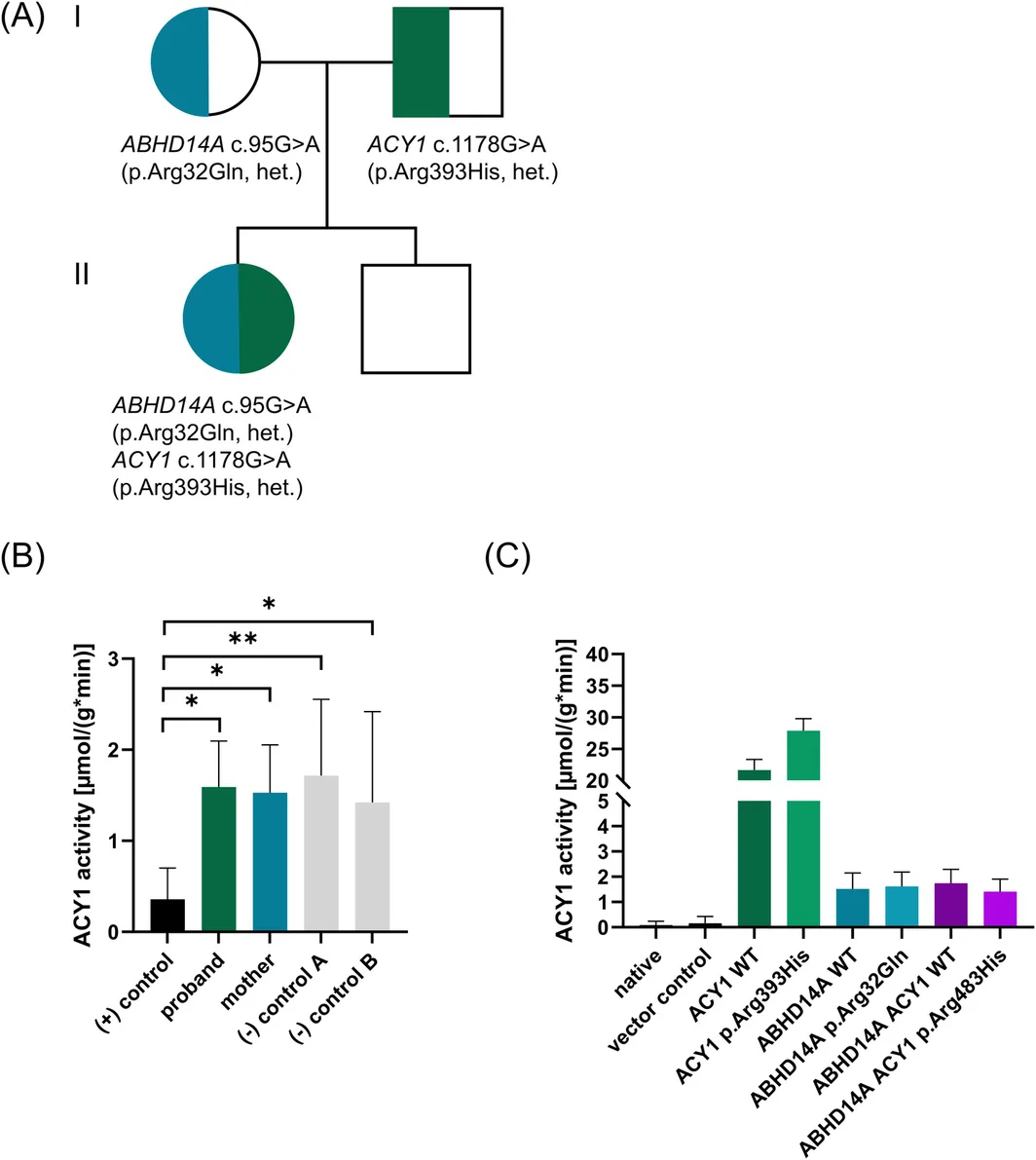
The patient‐derived variants in *ACY1* and *ABHD14A* do not affect ACY1 enzyme activity. (A) Schematic illustration of the family's pedigree. The variant in *ABHD14A* c.95G>A was inherited from the mother, the variant in *ACY1* c.1178G>A from the father. The patient carries both variants. (B) Variants in *ACY1* and *ABHD14A* do not affect ACY1 enzyme activity in EBV‐transformed patient‐derived lymphoblasts. Activity in patient and maternal cells is comparable to negative controls (−), while a marked reduction is observed in the positive control (+: cells derived from individual with known ACY1 deficiency; *n* = 6, mean ± SD). (C) ACY1 enzyme activity is increased in HEK293 cells overexpressing ACY1 or ACY1 p.Arg393His. ABHD14A constructs show moderate increases compared to native and vector controls (*n* = 3, mean ± SD). Statistical analysis was performed using repeated‐measures one‐way ANOVA followed by Dunnett's multiple comparison test, comparing each sample to the (+) control or the ACY1 WT in (B). In (C) all comparisons with ACY1 WT reached statistical significance at *p* < 0.0001, *p < 0.05, **p < 0.01.

The DNA extracted from peripheral blood of the entire family was analysed using WES. This analysis allowed the identification of two different variants: 3‐52023042‐G‐A(GRCh37) and 3‐52011912‐G‐A(GRCh37) in compound heterozygosity in the patient. The missense variant 3‐52023042‐G‐A(GRCh37), NM_000666.3:c.1178G>A (p.Arg393His) was found in exon 15 of the *ACY1* gene (Figure 1) and was inherited from the father (Figure 2A). This variant had been reported by Sass et al. in a proband affected by ACY1 deficiency in a compound heterozygote fashion together with c.1057C>T (p.Arg353Cys) [20]. Overexpression of the variant p.Arg393His in HEK293 cells yielded ACY1 enzyme activity comparable to that of the ACY1 wild‐type (WT), thus supporting a benign character of this variant [24]. As a second variant, the index case presented with a missense variant that was identified in exon 2 of the *ABHD14A* gene and maternally inherited: 3‐52011912‐G‐A(GRCh37), NM_015407.5:c.95G>A (p.Arg32Gln). This variant (rs17849626) was found at a frequency of 0.0354 in 1 613 784 control chromosomes in the GnomAD database and has not been reported to ClinVar. The variant received −10 ACMG points and is usually considered benign, while some of the computational scores listed at GeneBe suggest uncertainty or even pathogenicity. This variant has also been presented in an exome sequencing study on autism spectrum disorders [29].

After the WES result had been obtained, urinary organic acids of the patient were analysed in a qualitative approach by gas chromatography–mass spectrometry (GC–MS). *N*‐Acetylated derivates of glutamic acid, alanine, glycine, aspartic acid and valine were considered slightly elevated, although less pronounced than typically observed in ACY1 deficiency. We hypothesised that this biochemical finding could be explained by synergistic heterozygosity, with an *ACY1* variant in one allele and an *ABHD14A* variant in the other (Figure 2A). However, a limitation of this hypothesis is that the slight abnormality was observed only in a single urine sample, but not in a later obtained sample. In view of the specific question associated with the request for analysis, a bias regarding the attention to such signals in the first analysis cannot be completely excluded.

### 3.2 ACY1 Enzyme Activity Is Unaltered in Patient‐Derived Transformed Lymphocytes

Following Epstein–Barr virus‐mediated transformation of patient‐derived lymphocytes to lymphoblastoid cells, ACY1 enzyme activity was determined in homogenised cell sediments. The obtained patient‐derived lymphoblastoid cells yielded no decrease in ACY1 enzyme activity compared to negative controls and to lymphoblastoid cells derived from the patient's mother (Figure 2B). As generation of a lymphoblastoid cell line from his blood sample was not successful, ACY1 enzyme activity could not be assessed for cells of the patient's father, who was also heterozygous for the variant in *ACY1*. However, this *ACY1* variant, ACY1 p.Arg393His, has previously been reported not to lower ACY1 activity [24]. Hence, there is no evidence for ACY1 deficiency in any of the three family members studied. Thus, ACY1 enzyme activity was not substantially affected in patient‐derived cells.

### 3.3 ACY1 Enzyme Activity Is Unaltered in HEK293 Cells Overexpressing ABHD14A and Its Variant

To further investigate the potential functional consequences of the patient‐derived variants, overexpression studies were performed in HEK293 cells using pcDNA3.1(+) constructs representing an empty vector or encoding ACY1 WT and p.Arg393His, ABHD14A WT and p.Arg32Gln, ABHD14A ACY1 WT and p.Arg483His.

Overexpression of ACY1 WT or ACY1 p.Arg393His resulted in a 20‐ to 30‐fold increase in ACY1 enzyme activity compared with empty vector‐transfected or native HEK293 cells (Figure 2C). In contrast, overexpression of ABHD14A‐related constructs caused only a modest increase in ACY1 activity relative to controls, without major differences between the individual ABHD14A‐related constructs (Figure 2C). These findings indicate that neither the ACY1 nor the ABHD14A variants identified in the patient substantially affect ACY1 enzyme activity.

### 3.4 *ABHD14A* Transcript Levels Are Increased in HEK293 Cells Overexpressing ABHD14A and Its Variants

In addition to enzyme activity analyses, mRNA transcript levels were assessed in native HEK293 cells, cells overexpressing empty vector, ACY1 WT and p.Arg393His, ABHD14A WT and p.Arg32Gln, and ABHD14A ACY1 WT and p.Arg483His to quantitatively analyse gene expression. Therefore, total RNA was isolated, reverse transcribed and analysed by qPCR. For qPCR analysis, *ACY1*‐specific primers were designed to amplify a region shared by both the *ACY1* transcript and the *ABHD14A–ACY1* read‐through, which includes the full‐length *ACY1* sequence. Primers targeting *ABHD14A* and *ABHD14A–ACY1* were transcript‐specific (Table S1).

**FIGURE 3:**
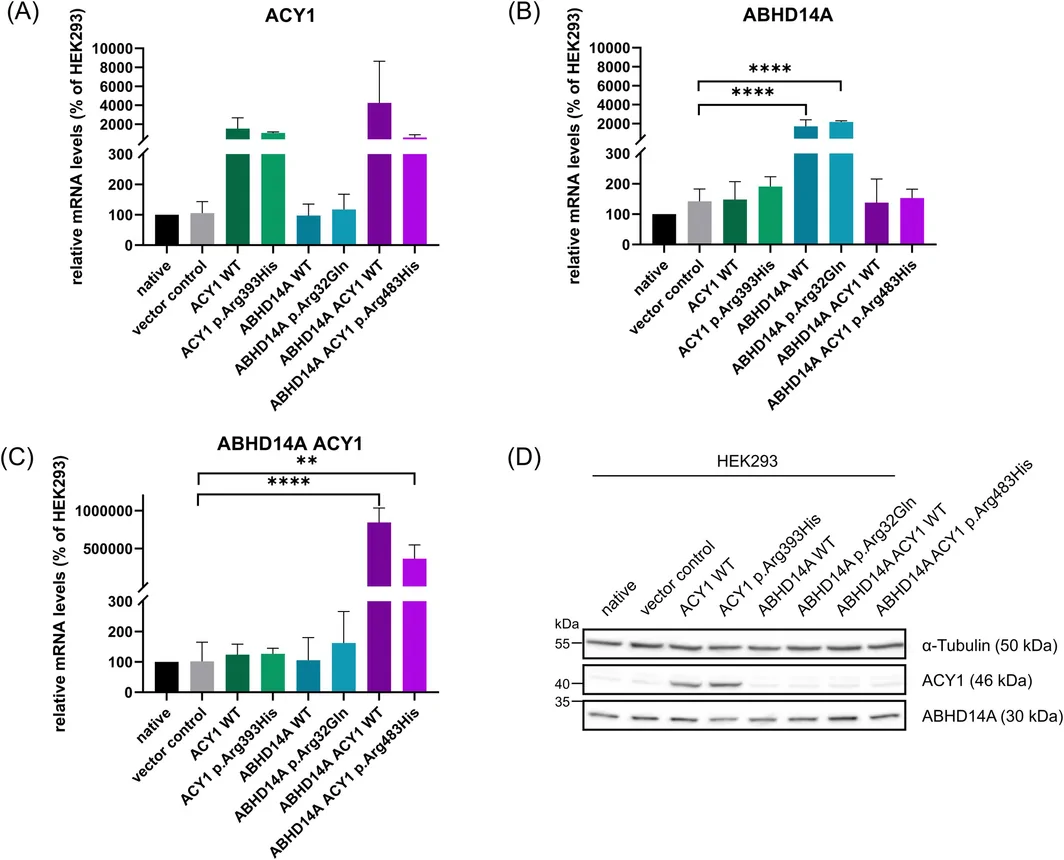
Relative mRNA levels and protein levels of *ACY1*, *ABHD14A*, and *ABHD14A*–*ACY1* variants in HEK293 cells. (A–C) qPCR analysis of cells overexpressing pcDNA3.1(+) constructs using gene‐specific primers. Transcript levels were normalised to GAPDH and expressed relative to native cells (100%) (*n* = 3; *n* = 2 for ACY1 p.Arg393His; mean ± SD). (D) Immunoblot analysis showing increased ACY1 protein levels upon overexpression of ACY1 WT and p.Arg393His, while ABHD14A protein levels are similar across all samples. A total of 30 μg of protein were subjected to SDS‐PAGE, α‐tubulin served as loading control. Statistical analysis was performed using repeated‐measures one‐way ANOVA followed by Dunnett's multiple comparison test, comparing each sample to the vector control in (A), (B), and (C): **p < 0.01, ****p < 0.0001. Non‐significant comparisons are not shown. Native, non‐transfected cells; vector control, empty pcDNA3.1(+); WT, wild type.

As expected, increased ACY1 enzyme activity in cells overexpressing *ACY1* WT and *ACY1* p.Arg393His went along with increased *ACY1* mRNA transcript levels. Moreover, overexpression of *ABHD14A–ACY1* and *ABHD14A–ACY1* p.Arg483His also resulted in increased *ACY1* transcript levels (Figure 3A). In contrast, *ABHD14A* transcript concentrations were selectively increased in cells overexpressing *ABHD14A* WT or *ABHD14A* p.Arg32Gln (Figure 3B). Analysis of the read‐through *ABHD14A–ACY1* transcripts revealed a pronounced upregulation exclusively in cells overexpressing *ABHD14A–ACY1* WT or *ABHD14A–ACY1* p.Arg483His (Figure 3C).

While increased *ACY1* transcript levels in *ACY1* WT and *ACY1* p.Arg393His overexpressing cells were accompanied by elevated enzyme activity; no corresponding increase in ACY1 enzyme activity was observed in cells overexpressing the *ABHD14A–ACY1* read‐through constructs, despite elevated *ACY1* transcript levels. Thus, qPCR analysis confirms transcriptional upregulation but does not distinguish between *ACY1* and *ABHD14A–ACY1*‐derived transcripts.

### 3.5 ABHD14A Protein Levels Are Similar Across All Overexpressed HEK293 Cells

To further validate the ACY1 enzyme activity and the detected mRNA transcript levels, immunoblot analysis was performed with the homogenates used for the enzyme activity tests. Protein expression of ACY1 and ABHD14A was analysed; α‐tubulin served as a loading control. Despite increased *ACY1* transcript levels in ABHD14A–ACY1 overexpressing cells, no corresponding increase in ACY1 enzyme activity was observed (Figure 2C). In view of this observation, it is unlikely (although not experimentally proven) that introduction of the ABHD14A p.Arg32Gln variant into an ACY1 WT read‐through would substantially contribute to ACY1 activity. Immunoblot analysis confirmed elevated ACY1 protein levels at the expected molecular weight of 46 kDa only in cells overexpressing ACY1 WT or ACY1 p.Arg393His. This is in agreement with the elevated ACY1 enzyme activity in these samples. In contrast, ABHD14A protein levels at 30 kDa appeared comparable across all analysed samples, and no read‐through protein was detectable on the immunoblot (Figure 3D).

## 4 Discussion

Variants in the *ACY1* gene have previously been reported to be the cause of ACY1 deficiency, an inborn error of metabolism first described two decades ago [2, 8]. The clinical phenotype of ACY1‐deficient patients is diverse, ranging from asymptomatic individuals to patients with neurological phenotypes [9, 20]. This clinical variability underlines the importance of functional validation of newly identified variants with uncertain significance in patients with suspected ACY1 deficiency, especially in the context of proposed other biological functions of ACY1. By using both patient‐derived lymphoblastoid cells and cells overexpressing the patient's variants in *ACY1* and *ABHD14A*, we demonstrate that ABHD14A p.Arg32Gln does not substantially affect ACY1 enzyme activity, and that ACY1 enzyme activity remains preserved in the studied patient. Furthermore, we demonstrate that no stable protein product of the ABHD14A–ACY1 read‐through transcript could be detected following overexpression.

In this study, we report a patient carrying variants in *ACY1* (c.1178G>A [p.Arg393His]) and *ABHD14A* (c.95G>A [p.Arg32Gln]) in trans. Both genes are genomically close and are known to form a naturally occurring read‐through transcript, suggesting a possible regulatory relationship. However, ACY1 enzyme activity in patient‐derived transformed lymphocytes remained unchanged compared to maternal cells and controls (Figure 2B). Similarly, overexpression of ACY1 p.Arg393His in HEK293 cells resulted in enzyme activity comparable to the WT ACY1 (Figure 2C). In accordance with this observation, it has been shown previously that overexpression of ACY1 p.Arg393His in HEK293 cells yields similar ACY1 enzyme activity to those obtained with the ACY1 WT [24]. This is in line with the fact that Arg 393 is not located within the catalytic core of ACY1 protein [24]. Taken together, our observations support the conclusion that the ACY1 p.Arg393His variant alone is unlikely to cause ACY1 enzyme deficiency.

Investigating the transcript levels of *ACY1*, *ABHD14A* and their variants after overexpression in HEK293 cells indicates that overexpression on the transcript level was successful. Increased ACY1 enzyme activity in ACY1‐overexpressing cells was accompanied by elevated *ACY1* transcript levels. In contrast, despite increased *ACY1* transcript levels in *ACY1‐ABHD14A*‐overexpressing cells, no corresponding increase in ACY1 enzyme activity was observed. This can be explained by the fact that the *ACY1* sequence within the *ABHD14A*–*ACY1* read‐through transcript is identical to the *ACY1* sequence, and the used primer pair amplifies this shared region as well. Although slight differences in enzyme activities were detectable following overexpression of ABHD14A, the read‐through construct and their variants, these effects remained modest and did not indicate a substantial functional impact on ACY1 activity.

Immunoblot analysis confirmed elevated ACY1 protein only in ACY1‐overexpressing cells, while ABHD14A protein levels appeared comparable across all samples. Importantly, the ACY1 antibody used in this study would also detect the read‐through. Nevertheless, no additional band corresponding to the expected size of the read‐through product was observed, suggesting the absence of a detectable ABHD14A–ACY1 protein. The epitope recognised by the ABHD14A antibody is absent in the read‐through transcript; detection would rely on the ACY1 antibody only, which supports this conclusion. Together, these findings support the hypothesis that the *ABHD14A*–*ACY1* read‐through transcript does not produce a stable functional protein.

In addition, *ABHD14A* and *ACY1* form a conjoined gene, for which transcript evidence has been reported [30]. The reported tissue expression of *ABHD14A*–*ACY1*, including foetal tissue RNA‐sequencing datasets [22], suggests that the read‐through may be subject to tissue‐ or developmental stage‐specific regulation. However, expression data alone does not indicate a physiological function of the transcript or its protein product. Consistent with our observations, sequence analysis in NCBI (NM_001316331.2) classifies the *ABHD14A*–*ACY1* read‐through transcript as non‐protein coding and has been annotated and predicted to be subject to nonsense‐mediated mRNA decay (NMD), due to an in‐frame stop codon located upstream of the coding sequence of the read‐through transcript. This is consistent with established NMD mechanisms triggered by premature stop codons and with the fact that ABHD14A–ACY1 read‐through protein has not been reported in the literature [31]. Notably, overexpression of the coding sequence alone did not result in a detectable protein, further supporting the hypothesis that it is a target for NMD. Due to the absence of detectable protein despite transcriptional upregulation in our experiments, it is likely that NMD, inefficient translation, or rapid degradation of an unstable protein takes place.

## 5 Conclusion

Considering the unchanged enzyme activity of ACY1 in both patient‐derived lymphoblastoid cells and HEK293 cells used for overexpression studies, we conclude that the patient's variants in *ACY1* and *ABHD14A* have no influence on the enzyme activity of ACY1. Interestingly, overexpression of *ABHD14A*, the read‐through and the corresponding variants did not yield detectable protein. This indicates NMD, inefficient translation, or the building of an unstable protein. Possible regulatory mechanisms regarding a potential protein expression of *ABHD14A*–*ACY1* warrant further investigation, while the benefit of its detection in routine testing is doubtful.

## Author Contributions

L.K. planned and performed most of the experiments, prepared the figures and participated in writing the manuscript. E.L.‐M., B.M.‐D., and M.P.P. performed genetic and phenotypic analysis of the patient. They initiated and coordinated the diagnostic procedure, to which A.R. contributed. Those four authors have read/critically revised the drafted manuscript. J.O.S. conceptualised and supervised the study and participated in writing the manuscript.

## Funding

Research by Jörn Oliver Sass is funded by the Deutsche Forschungsgemeinschaft (DFG, German Research Foundation), project numbers 514177501, 528562393‐FIP26, 538669957, and 558545467. Lil Klaas was supported by the Graduate Institute of H‐BRS and via the DFG project 528562393‐FIP26.

## Ethics Statement

The patient was recruited by the Undiagnosed Rare Diseases Patients Program, SpainUDP [32], at the Institute of Rare Diseases Research (IIER), Institute of Health Carlos III (ISCIII). Informed written consent was obtained from the legal representatives of the patient. This study was approved by the ISCIII Research Ethics Committee.

## Conflicts of Interest

The authors declare no conflicts of interest.

## Supporting information


**Table S1:** Sequences of used qPCR primers.

## Data Availability

Upon publication of this article, data from laboratory studies will be made available in Zenodo at https://doi.org/10.5281/zenodo.21096484.

## References

[1] National Center for Biotechnology Information (NCBI) , “ACY1, Gene ID: 95,” Gene Database, accessed May 11, 2026, https://www.ncbi.nlm.nih.gov/gene/95.

[2] J. O. Sass , V. Mohr , H. Olbrich , et al., “Mutations in *ACY1*, the Gene Encoding Aminoacylase 1, Cause a Novel Inborn Error of Metabolism,” American Journal of Human Genetics 78 (2006): 401–409. 10.1086/500563 PMC1380284 16465618

[3] H. Lindner , S. Höpfner , M. Täfler‐Naumann , M. Miko , L. Konrad , and K.‐H. Röhm , “The Distribution of Aminoacylase I Among Mammalian Species and Localization of the Enzyme in Porcine Kidney,” Biochimie 82 (2000): 129–137. 10.1016/s0300-9084(00)00191-7 10727768

[4] A. Biagini and A. Puigserver , “Sequence Analysis of the Aminoacylase‐1 Family. A New Proposed Signature for Metalloexopeptidases,” Comparative Biochemistry and Physiology Part B: Biochemistry and Molecular Biology 128 (2001): 469–481. 10.1016/s1096-4959(00)00341-9 11250542

[5] S. M. Birnbaum , L. Levintow , R. B. Kingsley , and J. P. Greenstein , “Specificity of Amino Acid Acylases,” Journal of Biological Chemistry 194 (1952): 455–470. 14927637

[6] M. Mitta , I. Kato , and S. Tsunasawa , “The Nucleotide Sequence of Human Aminoacylase‐1,” Biochimica et Biophysica Acta: Gene Structure and Expression 1174 (1993): 201–203. 10.1016/0167-4781(93)90116-u 8357837

[7] T. Giardina , A. Biagini , D. Massey‐Harroche , and A. Puigserver , “Distribution and Subcellular Localization of Acylpeptide Hydrolase and Acylase I Along the Hog Gastro‐Intestinal Tract,” Biochimie 81 (1999): 1049–1055. 10.1016/s0300-9084(99)00330-2 10575361

[8] R. Van Coster , N. E. A. Gerlo , T. G. Giardina , et al., “Aminoacylase I Deficiency: A Novel Inborn Error of Metabolism,” Biochemical and Biophysical Research Communications 338 (2005): 1322–1326. 10.1016/j.bbrc.2005.10.126 16274666

[9] J. O. Sass , J. Vaithilingam , C. Gemperle‐Britschgi , et al., “Expanding the Phenotype in Aminoacylase 1 (ACY1) Deficiency: Characterization of the Molecular Defect in a 63‐Year‐Old Woman With Generalized Dystonia,” Metabolic Brain Disease 31 (2016): 587–592. 10.1007/s11011-015-9778-6 26686503

[10] H. Chen , W. Wang , C. Xiao , D. Xia , F. Li , and S. Liu , “ACY1 Regulating PTEN/PI3K/AKT Signaling in the Promotion of Non‐Small Cell Lung Cancer Progression,” Annals of Translational Medicine 9 (2021): 1378. 10.21037/atm-21-3127 PMC8506526 34733930

[11] Z. Xu , Y. Hu , and Z. Yu , “Effect of the ACY‐1 Gene on HER2 and TRAIL Expression in Rectal Carcinoma,” Experimental and Therapeutic Medicine 22 (2021): 817. 10.3892/etm.2021.10249 PMC8193208 34131440

[12] Y. E. Miller , J. D. Minna , and A. F. Gazdar , “Lack of Expression of Aminoacylase‐1 in Small Cell Lung Cancer. Evidence for Inactivation of Genes Encoded by Chromosome 3p,” Journal of Clinical Investigation 83 (1989): 2120–2124. 10.1172/JCI114125 PMC303939 2542383

[13] A. S. M. Moin , T. Sathyapalan , S. L. Atkin , and A. E. Butler , “Diagnostic and Prognostic Protein Biomarkers of β‐Cell Function in Type 2 Diabetes and Their Modulation With Glucose Normalization,” Metabolites 12 (2022): 196. 10.3390/metabo12030196 PMC8950787 35323639

[14] National Center for Biotechnology Information (NCBI) , “ABHD14A, Gene ID: 25864,” Gene Database, accessed May 11, 2026, https://www.ncbi.nlm.nih.gov/gene/25864/.

[15] C. C. Lord , G. Thomas , and J. M. Brown , “Mammalian Alpha Beta Hydrolase Domain (ABHD) Proteins: Lipid Metabolizing Enzymes at the Interface of Cell Signaling and Energy Metabolism,” Biochimica et Biophysica Acta: Molecular and Cell Biology of Lipids 1831 (2013): 792–802. 10.1016/j.bbalip.2013.01.002 PMC4765316 23328280

[16] D. L. Ollis , E. Cheah , M. Cygler , et al., “The *α*/*β* Hydrolase Fold,” Protein Engineering 5 (1992): 197–211. 10.1093/protein/5.3.197 1409539

[17] K. Vaidya , G. Rodrigues , S. Gupta , et al., “Identification of Sequence Determinants for the abhd14 Enzymes,” Proteins 93 (2025): 255–266. 10.1002/prot.26632 37974539

[18] J. Hoshino , J. Aruga , A. Ishiguro , and K. Mikoshiba , “ *Dorz1*, a Novel Gene Expressed in Differentiating Cerebellar Granule Neurons, Is Down‐Regulated in *Zic1*‐Deficient Mouse,” Molecular Brain Research 120 (2003): 57–64. 10.1016/j.molbrainres.2003.10.004 14667578

[19] J. P. Casey , T. Magalhaes , J. M. Conroy , et al., “A Novel Approach of Homozygous Haplotype Sharing Identifies Candidate Genes in Autism Spectrum Disorder,” Human Genetics 131 (2012): 565–579. 10.1007/s00439-011-1094-6 PMC3303079 21996756

[20] J. O. Sass , H. Olbrich , V. Mohr , et al., “Neurological Findings in Aminoacylase 1 Deficiency,” Neurology 68 (2007): 2151–2153. 10.1212/01.wnl.0000264933.56204.e8 17562838

[21] A. Tylki‐Szymanska , W. Gradowska , A. Sommer , et al., “Aminoacylase 1 Deficiency Associated With Autistic Behavior,” Journal of Inherited Metabolic Disease 33, no. S3 (2010): S211–S214. 10.1007/s10545-010-9089-3 20480396

[22] National Center for Biotechnology Information (NCBI) , “ABHD14A‐ACY1, Gene ID: 100526760,” Gene Database, accessed May 27, 2026, https://www.ncbi.nlm.nih.gov/gene/100526760.

[23] A. Korwitz‐Reichelt , D. Schulke , M. Walter , and J. O. Sass , “Human d‐Glycerate Kinase, Encoded by *GLYCTK* and Deficient in d‐Glyceric Aciduria, Is a Mitochondrial Enzyme,” Journal of Inherited Metabolic Disease 49 (2026): e70119. 10.1002/jimd.70119 41330740

[24] A. Sommer , E. Christensen , S. Schwenger , et al., “The Molecular Basis of Aminoacylase 1 Deficiency,” Biochimica et Biophysica Acta: Molecular Basis of Disease 1812 (2011): 685–690. 10.1016/j.bbadis.2011.03.005 21414403

[25] S. Laurie , D. Piscia , L. Matalonga , et al., “The RD‐Connect Genome‐Phenome Analysis Platform: Accelerating Diagnosis, Research, and Gene Discovery for Rare Diseases,” Human Mutation 43 (2022): 717–733. 10.1002/humu.24353 PMC9324157 35178824

[26] P. Stawiński and R. Płoski , “Genebe.Net: Implementation and Validation of an Automatic ACMG Variant Pathogenicity Criteria Assignment,” Clinical Genetics 106 (2024): 119–126. 10.1111/cge.14516 38440907

[27] O. H. Lowry , N. J. Rosebrough , A. L. Farr , and R. J. Randall , “Protein Measurement With the Folin Phenol Reagent,” Journal of Biological Chemistry 193 (1951): 265–275. 14907713

[28] K. J. Livak and T. D. Schmittgen , “Analysis of Relative Gene Expression Data Using Real‐Time Quantitative PCR and the 2^−ΔΔ*C*T^ Method,” Methods 25 (2001): 402–408. 10.1006/meth.2001.1262 11846609

[29] H. N. Cukier , N. D. Dueker , S. H. Slifer , et al., “Exome Sequencing of Extended Families With Autism Reveals Genes Shared Across Neurodevelopmental and Neuropsychiatric Disorders,” Molecular Autism 5 (2014): 1. 10.1186/2040-2392-5-1 PMC3896704 24410847

[30] T. Prakash , V. K. Sharma , N. Adati , et al., “Expression of Conjoined Genes: Another Mechanism for Gene Regulation in Eukaryotes,” PLoS One 5 (2010): e13284. 10.1371/journal.pone.0013284 PMC2953495 20967262

[31] Y.‐F. Chang , J. S. Imam , and M. F. Wilkinson , “The Nonsense‐Mediated Decay RNA Surveillance Pathway,” Annual Review of Biochemistry 76 (2007): 51–74. 10.1146/annurev.biochem.76.050106.093909 17352659

[32] E. López‐Martín , B. Martínez‐Delgado , E. Bermejo‐Sánchez , J. Alonso , The SpainUDP Network , and M. Posada , “SpainUDP: The Spanish Undiagnosed Rare Diseases Program,” International Journal of Environmental Research and Public Health 15 (2018): 1746. 10.3390/ijerph15081746 PMC6121381 30110963

