# Supplementary material for: A Variant in Abhydrolase Domain Containing 14A (ABHD14A) and the Suspicion of Aminoacylase 1 (ACY1) Deficiency
Source: JIMD Rep. 2026 Sep 18;67(5):e70124. doi: 10.1002/jmd2.70124 (PMC13588756; doi:10.1002/jmd2.70124)
Supplement: Supplementary file 1 [file JMD2-67-e70124-s001.docx]

**Supplementary** **Table 1: Sequences of used qPCR Primers.**

| **Gene** | **Forward Primer 5’-3’** | **Reverse Primer 5’-3’** |
| --- | --- | --- |
| *GAPDH* | TGGGGAAGGTGAAGGTCGGA | TCCCGTTCTCAGCCTTGACG |
| *ACY1* | GCCACCGGTTCCCCAGAACC | GCTGGTAACCCGCACCCACC |
| *ABHD14A* | GAGGTACAGAATGCCGTGTTGGTGAG | AGCTCTCCATACAGGATAAGGGTTGGAGTC |
| *ABHD14A-ACY1* | TCAACCAGGCACACAGGGTGGA | GGACCCTTGCTGGTCATGGCG |
